# The effect of nanobased irrigants on the root canal dentin microhardness: an *ex-vivo* study

**DOI:** 10.1186/s12903-023-03298-z

**Published:** 2023-08-19

**Authors:** Safoora Sahebi, Hossein Mofidi, Abbas Abbaszadegan, Ahmad Gholami, Fateme Eskandari

**Affiliations:** 1 Department of Endodontics, School of Dentistry, Shiraz University of Medical Sciences, Shiraz, Fars Iran; 2 Endodontist, Department of Endodontics, School of Dentistry, Shiraz University of Medical Sciences, Shiraz, Iran; 3 Biotechnology Research Center, Shiraz University of Medical Sciences, Shiraz, Fars Iran; 4 Pharmaceutical Sciences Research Center, Shiraz University of Medical Sciences, Shiraz, Fars Iran; 5 Department of Pharmaceutical Biotechnology, School of Pharmacy, Shiraz University of Medical Sciences, Shiraz, Fars Iran; 6 School of Dentistry, Shiraz University of Medical Sciences, Ghasrdasht Street, Shiraz, 71956-15878 Iran

**Keywords:** 1-dodecyl-3-methylimidazolium, Dentin, Endodontics, Hardness, Nanoparticles, Nanotechnology, Root canal irrigants, Root canal therapy, Silver, Zinc oxide

## Abstract

**Background:**

Given the favorable antimicrobial properties of zinc oxide (ZnONPs), standard silver (AgNPs), and imidazolium-based silver (Im-AgNPs) nanoparticles, this study aimed to evaluate their influence on the microhardness of root canal dentin.

**Methods:**

In this experimental study, 40 mandibular premolars were decoronated at the cementoenamel junction and longitudinally sectioned into halves to create 80 specimens. They were randomly allocated to 5 groups (n = 16) and irrigated with ZnONPs, AgNPs, Im-AgNPs, NaOCl, or normal saline (as the negative control) for 15 min. The Vickers Hardness Number (VHN) was measured on each root canal third before and after being soaked in irrigants. Statistical analysis was performed using paired t-test, one-way ANOVA, and post hoc Tukey’s test (α = 0.05).

**Results:**

Im-AgNPs and ZnONPs irrigants improved the microhardness of root dentin, whereas, AgNPs and NaOCl decreased it. ZnONPs yielded the highest VHN at the coronal third (P˂0.001), while the Im-AgNPs provided the highest VHN at the middle and apical thirds (P˂0.001). The AgNPs group showed the lowest VHN at the apical third.

**Conclusions:**

The irrigants containing Im-AgNPs and ZnONPs significantly enhanced the root dentin microhardness. However, the use of AgNPs resulted in decreased microhardness.

## Background

The foremost aim of root canal treatment is to eradicate microorganisms and their by-products from the root canal system [1, 2]. Although various irrigants have been recommended over the years to enhance root canal disinfection and endodontic treatment success [3, 4], no single solution has yet met all the criteria of an ideal endodontic irrigant. Currently, sodium hypochlorite (NaOCl) is considered the gold standard due to its high efficacy AgNPs against bacteria and biofilm, as well as its ability to dissolve organic debris [5–7]. However, it has several drawbacks including an unpleasant taste, toxicity, incomplete elimination of the smear layer, and negative effects on dentin flexural strength [3, 8]. Consequently, researchers continue to seek alternative irrigants with greater efficacy and fewer side effects [5].

Nanotechnology, a brilliant achievement of the last decade, has revolutionized science research and development [9, 10]. Some nanoparticles, such as silver (AgNPs), possess remarkable antimicrobial properties [11, 12], even AgNPs against drug-resistant pathogens such as *E. faecalis* [13, 14], which is due in part to their nano size, which enables them to penetrate microorganisms’ cell membrane more effectively. Imidazolium-coated silver (Im-AgNPs) nanoparticles, engineered specifically for endodontic applications, have gained popularity as potential root canal irrigants due to their antimicrobial properties, biocompatibility, and other promising features [15–17]. Abbaszadegan et al. [18] found that positively-charged Im-AgNPs nanoparticles had a significant impact on the antibacterial activity of this novel irrigant AgNPs against *E. faecalis*. Moreover, these nanoparticles resulted in higher surface roughness and were biocompatible and cytocompatible with L929 cells, while exhibiting low cytotoxicity compared to commonly used irrigants such as sodium hypochlorite and chlorhexidine [18–21].

Zinc oxide (ZnONPs) is another metal nanoparticle with promising antibacterial activity even against antimicrobial resistant microorganisms [22] and favorable properties for use in Endodontics, as it exhibits selective toxicity against dental bacterial pathogens with minimal effect on human cells [23, 24]. In addition, ZnONPs nanoparticles have been reported to improve the physical and chemical features of the Grossman sealer [25]. Some nanobased irrigants (e.g. AgNPs, ZnONPs, and titanium dioxide) have been found to enhance the fracture resistance of endodontically treated teeth [26].

Some root canal irrigants, such as NaOCl and ethylenediaminetetraacetic acid (EDTA), can affect the structural characteristics of dentin like microhardness, and consequently impact the quality of obturation and coronal restoration [27–29]. Given the absence of a similar study, the current study aimed to assess the microhardness of root canal dentin after treatment with standard AgNPs, Im-AgNPs, and ZnONPs nanoparticles compared with 2.5% NaOCl and normal saline. The null hypothesis was that these irrigants would not significantly affect the dentin microhardness.

## Methods

### Sample size calculation

Following previous research [30], a power calculation was conducted by using the chi-square test family and variance statistical test (G*Power 3.1 software; Heinrich Hein University, Dusseldorf, Germany) with a significance level of α = 0.05 and a power of ß=0.95. The minimum sample size was determined to be 12 per group.

### Preparation of tooth samples

This *ex-vivo* experimental study was ethically approved by the Ethics Committee of Shiraz University of Medical Sciences (IR.SUMS.DENTAL.REC.1400.043). It was performed in full accordance with ethical principles, including the World Medical Association Declaration of Helsinki (version 2008). The study design for the present experiment was based on the methodology of Saha et al. [31]. Forty mandibular premolars extracted for orthodontic purposes were collected with written informed consent from patients. The teeth were cleaned of any soft tissue or calculus deposits and stored in 0.1% thymol solution under refrigeration until used. Proximal view radiographs were taken to confirm the presence of a single patent canal. Teeth with root caries, cracks, curved canals, a history of endodontic treatment, internal resorption, or calcification were excluded. The specimens were decoronated at the cementoenamel junction using a double-faced diamond disc (Microdont, LDA, Brazil) at low speed under water cooling to ensure a uniform sample length of 15 ± 1 mm root length.

### Specimen preparation

Plastic cubes (with dimensions of 3 × 3 × 3 cm^3^) were filled with transparent polyester material, and the extracted teeth were inserted with the dentinal surface fronting outward. The resulting blocks were then longitudinally sectioned under water cooling, resulting in 80 dentin halves. These sections were examined at 4× magnification using a Dino-Lit USB Digital Microscope (AM3111-0.3 MP; Dino-Lit, Vietnam) to identify and exclude any cracked teeth. Surface scratches were then ground-polished under water coolant using a series of ascending grades of carbide abrasive papers (500, 800, 1000, and 1200 grit) (Bigo, Dent Product, Germany), followed by a final polish with a 0.1-mm alumina suspension on a rotary felt disc (Microdont, LDA, Brazil) to achieve a smooth glossy mirror-like surface.

The specimens were randomly (simple randomization) allocated to 5 groups (n = 16 per group) to receive irrigation with any of standard AgNPs, Im-AgNPs, 0.1% ZnONPs (Nano-Mavad-Gostaran Pars, Tehran, Iran), 2.5% NaOCl (Sigma-Aldrich Corporation, St Louis, MO, USA), or normal saline (as the negative control).

### Synthesis of standard AgNPs nanoparticles

Silver nitrate powder was purchased from Merck, Darmstadt, Germany. The synthesis protocol was as follows; 50 mL of 1 mM silver nitrate (AgNO_3_) solution was mixed with 1.0 mL of 1 mM trisodium citrate in a 100-mL volumetric flask and stirred vigorously. After 15 min, 50 µL of 100 mM sodium borohydride solution was rapidly added to this mixture solution. The color of the solution initially turned pale yellow and later turquoise. When the solution cooled to room temperature, it was centrifuged at 5000 rpm for 10 min. The supernatant was discarded and the pellet was air-dried in an incubator [15, 32–35].

### Synthesis of Im-AgNPs irrigant

Twenty mL of 1-dodecyl-3-methylimidazolium chloride (6.2 mM) (Sigma-Aldrich corporation, St Louis, MO, USA) was mixed with an aqueous solution of AgNPsNO_3_ (0.01 M) and vigorously stirred. An aqueous solution of 0.4 M NaBH_4_ was instantly added dropwise to the mixture overnight to obtain a golden-colored solution. The colloidal solution was centrifuged for 20 min to eliminate unreacted ions. The initial concentration of the nanoparticle solution before the experiment was 1024 µg/mL.

### Synthesis of ZnONPs

To synthesize the ZnONPs solution, the ZnONPs powder (Nano-Mavad-Gostaran Pars, Tehran, Iran) was dissolved in phosphate-buffered saline solution and stirred on a magnetic stirrer for 24 h. The solution was then sonicated to ensure homogeneity.

### Characterization of nanoparticles

The nanoparticles were subjected to multiple characterization techniques, including ultraviolet-visible spectroscopy (UV-2550, Shimadzu, Japan), transmission electron microscopy ([TEM], Zeiss-EM10C-100 KV, Germany), X-ray diffraction spectroscopy ([XRD], Siemens D5000 diffractometer, Karlsruhe, Germany), field emission scanning electron microscope (FESEM, Sigma VP, ZEISS, Germany, Energy-dispersive X-ray spectroscopy ([EDS], FESEM, Sigma VP, ZEISS, Germany), and Zeta potential measurements (Zeta-DLS; Zetasizer, Malvern, England). The Im-AgNPs were lyophilized in the same way as the dried standard silver nanoparticles for above mentioned characterization studies.

### Measurement of dentin microhardness

To measure the pre-treatment microhardness, each sample was individually mounted on the stage of the Vickers microhardness testing machine (FM 700, Future-T,ech, Tokyo, Japan) and three indentations were marked at each root canal third (middle, apical, and coronal) by using a Vickers diamond indenter at a 300 gr load and a 20-second dwell time [31]. To standardize the measurements, indentations were made on the dentin surface at approximately 200 μm from the dentin-canal interface. The Vickers hardness value was calculated by dividing the applied load by the area of the sloping faces of the indentations. The resulting impression of the two diagonals was observed through an optical microscope, the average length of the two diagonals was measured by the built-in scaled micrometer and converted into the Vickers hardness number (VHN) using the following formula:

#### VHN (HV) = 1.854(F/D^2^)

The formula’s constant value was calculated based on the specific geometry of the indenter, where F represents the applied load (grams) and D represents the diagonal of the indentation (µm) [36].

For the pre-treatment VHN, three indentations were marked on each root canal third (middle, apical, and coronal), and the average VHNs was calculated for each third. The specimens were randomly allocated into 5 groups (n = 16) and immersed in 5 mL of standard AgNPs, Im-AgNPs, ZnONPs, and NaOCl nanobased irrigants, as well as normal saline as the negative control, for 15 min. Following immersion, the specimens were rinsed with normal saline, and dried, and the post-treatment VHN was measured using the same method as for the pre-treatment VHN.

### Statistical analysis

The data were statistically analyzed by using SPSS software (version 16, SPSS INC., Chicago, IL, USA) through one-way ANOVA (for intergroup comparison), Tukey’s post hoc test (for pairwise comparison of the groups regarding the mean hardness), and paired t-test (to compare the effect of irrigants). P values ˂0.05 were considered significant.

## Results

### Synthesis and characterization of nanobased irrigants

#### Ultraviolet-visible spectroscopy

**Fig. 1:**
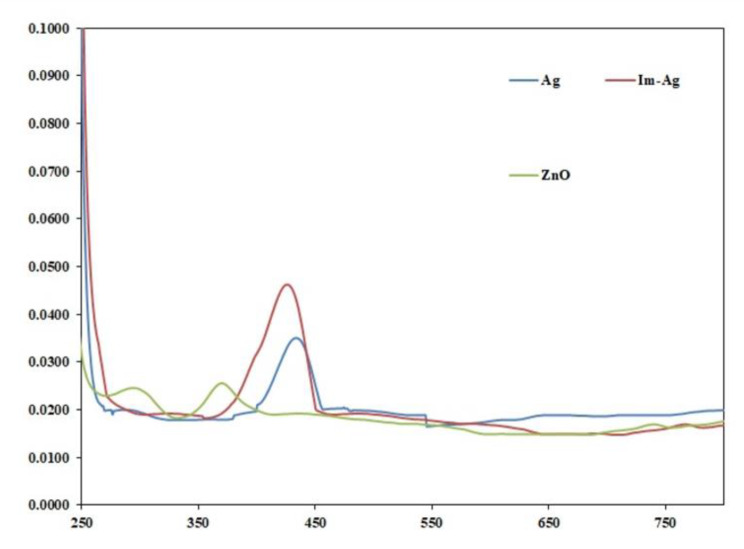
The UV-visible spectrum of AgNPs, Im-AgNPs, and ZnONPs irrigants

Figure 1 shows the UV-visible spectra of AgNPs, Im-AgNPs, and ZnONPs irrigants. The spectrum of AgNPs and Im-AgNPs exhibited characteristic peaks at around 440 nm and 428 nm, respectively, confirming the formation of AgNPs nanoparticles resulting from the reduction of AgNPs^+^ ions to AgNPs^0^. Previous findings indicated that the absorption bands at around 400 nm in the UV-visible spectrum are attributed to the spherical AgNPs nanoparticles [37]. The ZnONPs group showed a characteristic peak at a wavelength of 376 nm, indicating the formation of ZnONPs nanoparticles from zinc nitrate solution. This was further supported by the observed color alteration from dark brown to light brown due to the synthesis of ZnONPs nanoparticles. This wavelength is consistent with the reported characteristic peak of ZnONPs in the literature [38, 39].

#### Energy-dispersive X-ray spectroscopy

**Fig. 2:**
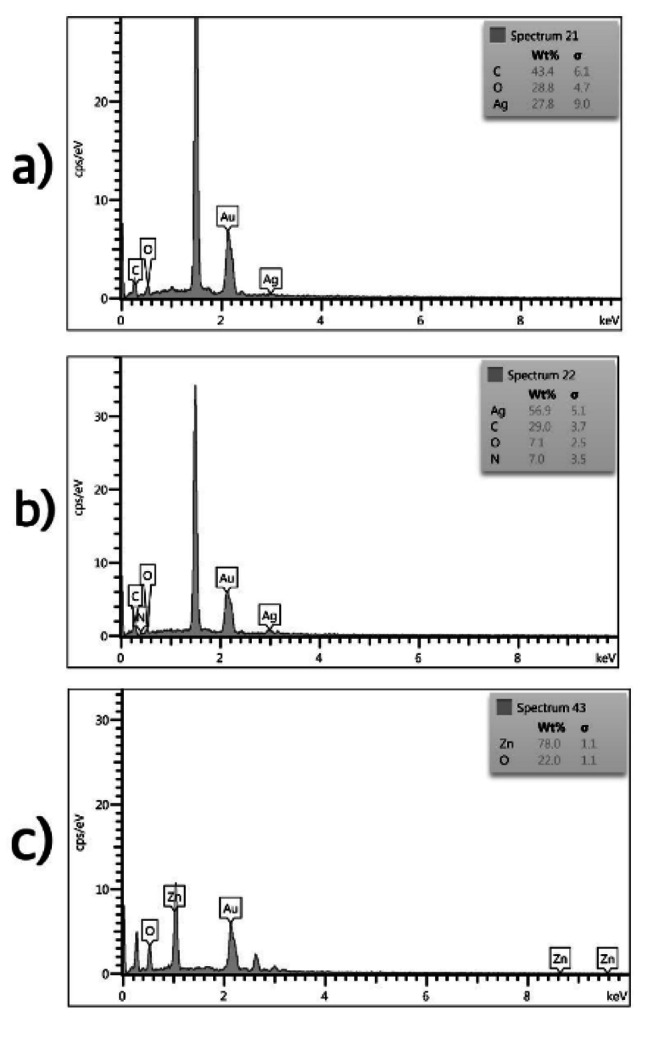
The EDS analysis of synthesized nanoparticles; (a) AgNPs (b) Im-AgNPs (c) ZnONPs

The EDS analysis results of the nanoparticles showed different elemental compositions for each group. The AgNPs nanoparticles had carbon (43.4%), oxygen (28.8%), and AgNPs (27.8%) elements. The Im-AgNPs had a strong AgNPs signal (56.9%), as well as carbon (29%), oxygen (7.1%), and nitrogen (7%) elements. The EDS analysis of ZnONPs nanoparticles demonstrated the presence of oxygen (22%) and zinc elements (78%), confirming the formation of zinc oxide (Fig. 2).

#### Zeta potential

Based on the analysis of Zeta potential, the surface charge was + 16.47 in AgNPs, + 50.90 in Im-AgNPs, and − 7.44 in ZnONPs.

#### X-ray diffraction analysis

**Fig. 3:**
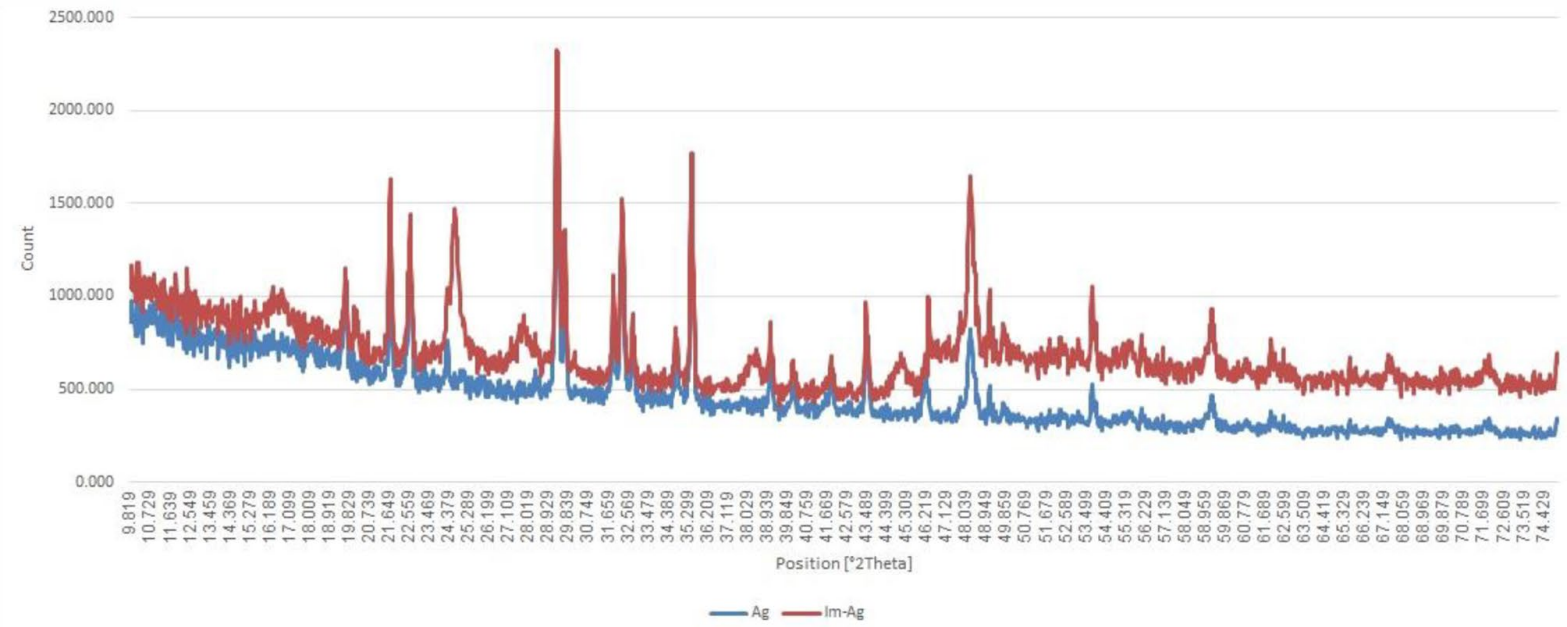
The XRD patterns of AgNPs and Im-AgNPs

**Fig. 4:**
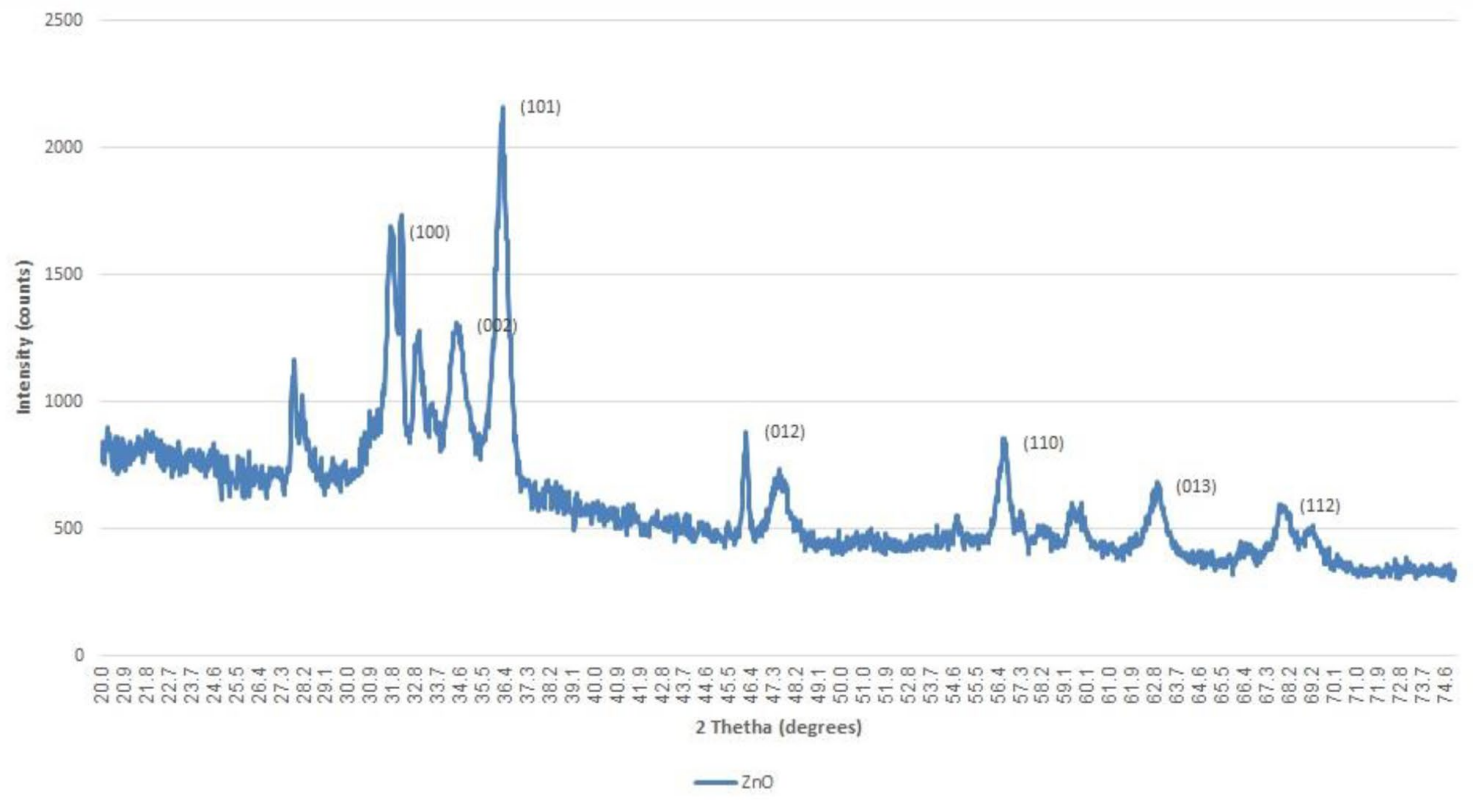
The XRD patterns of ZnONPs

The XRD patterns of AgNPs and Im-AgNPs nanoparticles validated their crystalline nature, with diffraction peaks at 2θ = 39.113°, 43.488°, and 65.59° (Fig. 3), which were consistent with previous studies [37, 40–42]. Similarly, the XRD pattern of ZnONPs nanoparticles confirmed their crystalline nature, with peaks (and Miller indices) at 2θ = 32.2° (100), 34.4° (002), 36.3° (101), 46.2° (012), 56.7° (110), 63° (013), and 67.9° (112) (Fig. 4), which was in agreement with Lodhi et al.‘s study [43]. The absence of any additional peaks in the XRD pattern indicated the high purity of the product.

#### Transmission electron microscopy

**Fig. 5:**
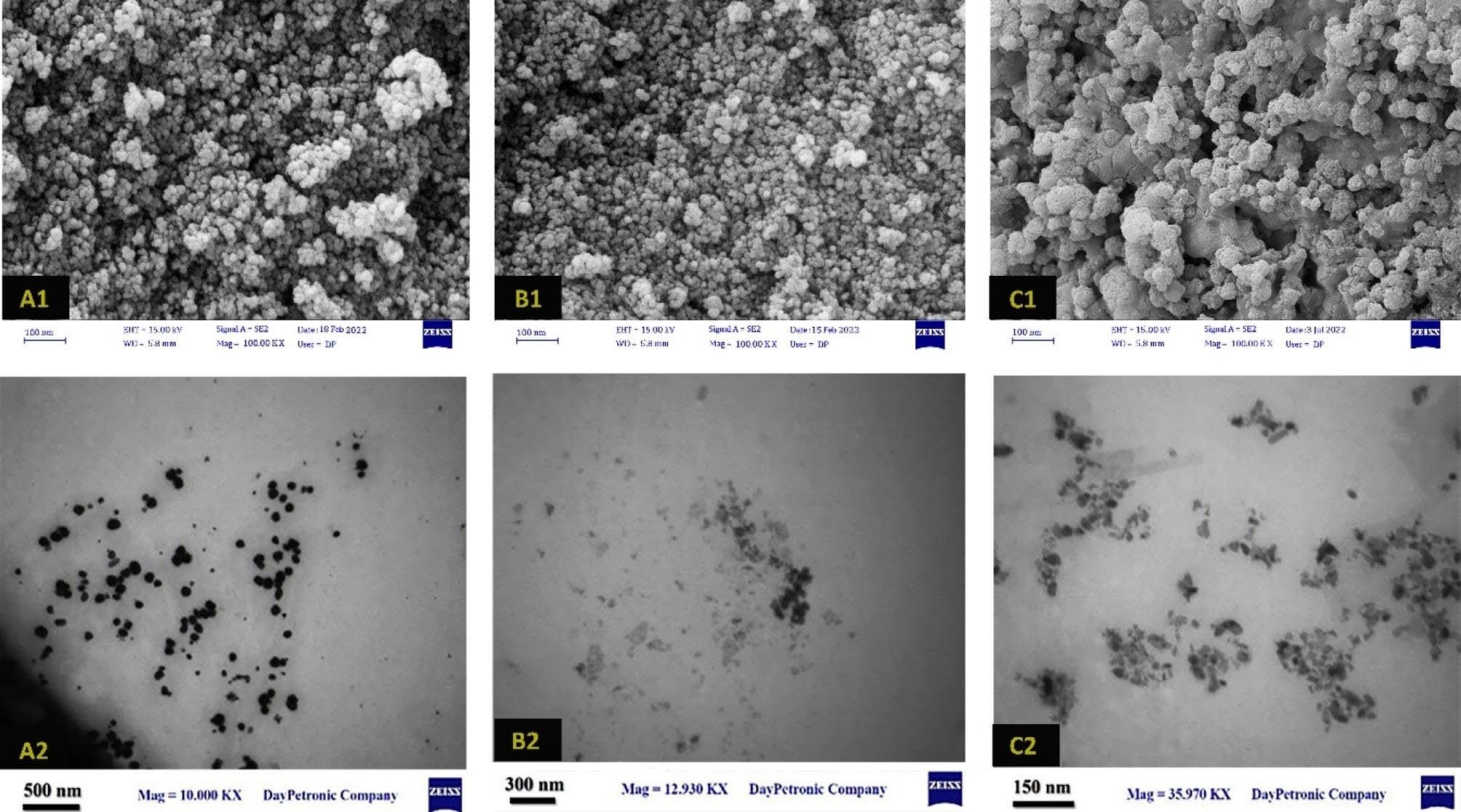
The FESEM micrographs of the synthesized nanoparticles: A1) AgNPs B1) Im-AgNPs C1) ZnONPs/ The TEM images of the synthesized nanoparticles: A2) AgNPs B2) Im-AgNPs C2) ZnONPs

The TEM images showed that the synthesized AgNPs, Im-AgNPs, and ZnONPs nanoparticles were all spherical in shape, confirming the results obtained from UV-visible spectroscopy (Fig. 5). The average size of 50 isolated particles from each TEM image was measured to calculate their mean sizes, which were 24.83 ± 11.96 nm in AgNPs, 27.17 ± 15.62 nm in Im-AgNPs, and 18.83 ± 6.25 nm in ZnONPs nanoparticles. The FESEM graphs of the synthesized NPs are demonstrated in Fig. 5. The average size of AgNPs, Im-AgNPs, and ZnONPs was calculated 10.74 nm, 11.17 nm, and 15.12 nm from FESEM micrographs.

#### Microhardness assessment

The Kolmogorov-Smirnov test showed that the data were normally distributed (P˃0.05). One-way ANOVA and post hoc Tukey’s test demonstrated that the pre-treatment VHNs were not significantly different among the groups (P˃0.05). The pre- and post-treatment VHNs were not significantly different in the control group (P˃0.05). Regardless of the root canal thirds, the VHN was the highest in the ZnONPs group, followed by Im-AgNPs, normal saline, NaOCl, and standard AgNPs groups, respectively (Table 1).

**Table 1:** Mean ± SD of root canal dentin microhardness following irrigation by experimental irrigants regardless of root canal thirds

Groups	Mean ± SD
**Standard AgNPs**	53.17 ± 9.18
**Im-AgNPs**	66.79 ± 5.53
**ZnONPs**	67.81 ± 9.10
**2.5% NaOCl**	59.67 ± 7.12
**Normal saline**	63.22 ± 8.57

Immersion in standard AgNPs nanobased irrigant significantly decreased the VHN compared to normal saline in the apical and coronal thirds (P˂0.001). Conversely, immersion in Im-AgNPs significantly increased the VHN in the middle and apical thirds (P˂0.05). Immersion in standard AgNPs nanobased irrigant and NaOCl reduced the VHNs in the coronal and apical root canal thirds, with statistical significance observed in the coronal third of the root canal (P˂0.001). In the middle third, no significant difference was observed following immersion in standard AgNPs and NaOCl (P˂0.05). On the other hand, ZnONPs nanobased irrigant increased the VHN, particularly in the coronal third (P˂0.001). Pairwise comparison of post-treatment VHNs in different root canal thirds showed that the highest VHNs at the coronal and middle thirds were in the ZnONPs group (P˂0.001); while the highest VHN in the apical thirds was in the Im-AgNPs group (P˂0.001) (Table 2).

**Table 2:** Comparison of the pre- and post-treatment VHNs in each root canal third (paired t-test)

	Coronal			Middle			Apical		
Group	Pre-treatment	Post-treatment	P value	Pre-treatment	Post-treatment	P value	Pre-treatment	Post-treatment	P value
**Standard AgNPs**	60.56 ± 9.31	48.95 ± 8.18^d*^	˂0.001	55.08 ± 9.22	55.44 ± 8.41^d*^	0.888	59.66 ± 12.01	55.08 ± 9.22^bd*^	0.318
**Im-AgNPs**	68.39 ± 11.63	64.56 ± 5.58^ac^	0.245	57.07 ± 10.40	67.25 ± 6.17^ac^	˂0.001	59.54 ± 9.71	68.54 ± 4.24^a^	˂0.001
**ZnONPs**	64.24 ± 12.02	72.83 ± 9.07^a^	˂0.001	65.06 ± 9.76	68.74 ± 6.70^a^	0.182	60.23 ± 12.44	61.86 ± 8.15^ab^	0.630
**2.5% NaOCl**	71.26 ± 17.12	57.54 ± 6.48^bc^	˂0.001	59.68 ± 15.91	60.55 ± 5.14^ cd^	0.850	65.20 ± 9.65	60.94 ± 9.11^ab^	0.317
**Normal saline**	65.66 ± 7.76	67.95 ± 8.12^a^	0.304	60.43 ± 9.71	62.27 ± 7.61^ac^	0.603	59.87 ± 12.19	59.45 ± 8.24^bc^	0.909
**P value**		˂0.001			˂0.001			0.03	

*Different superscript lowercase letters in a column indicate a statistically significant difference

## Discussion

The UV-visible spectrum of AgNPs and Im-AgNPs showed characteristic peaks around 440 nm and 428 nm, respectively, which confirms the formation of AgNPs through the reduction of AgNPs^+^ ions to AgNPs^0^. According to earlier research, the spherical AgNPs nanoparticles contribute to absorption bands at around 400 nm in the UV-visible spectrum [37]. The 376-nm wavelength seen in the UN-visible spectrum is a characteristic peak of the ZnONPs group. This peak indicates the formation of ZnONPs nanoparticles from the zinc nitrate solution used in the synthesis, which was also confirmed by the visible color change from dark brown to light brown. The reported wavelength is consistent with previous literature on the characteristic peak of ZnONPs [38, 39].

The XRD analysis confirmed the crystalline nature of AgNPs and Im-AgNPs, as evidenced by the diffraction peaks at 2θ = 39.113°, 43.488°, and 65.59°; which were consistent with previous studies [37, 40–42]. The XRD pattern for ZnONPs showed characteristic peaks (and Miller indices) at various 2θ angles including 32.2° (100), 34.4° (002), 36.3° (101), 46.2° (012), 56.7° (110), 63° (013), and 67.9° (112) (Fig. 4), which were in agreement with Lodhi et al.‘s findings [43]. The absence of any extra peaks in this XRD pattern indicated the high purity of the product.

The null hypothesis was partially rejected as some nanobased irrigants significantly affected the microhardness of root canal dentin. The findings revealed that the application of AgNPs as root canal irrigant negatively influenced root dentin microhardness. Moreover, VHNs following the usage of Im-AgNPs significantly increased at the middle and apical third, while its raise at the coronal third was not significant. Besides, irrigation of the samples with AgNPs at the coronal third remarkably decreased the microhardness values. Final flushing of the root canals with ZnONPs solution improved the values of microhardness at all root canal thirds, but only at the coronal third its improvement was statistically significant. Sodium hypochlorite significantly reduced VHN at the coronal third.

The success of endodontic treatment depends on the quality and method of chemomechanical preparation, disinfection, and 3-dimensional obturation of the root canals [44–46]. Endodontic irrigants can alter the chemical composition of dentin by affecting its organic and inorganic phases, which can lead to a reduction in dentin microhardness and an increase in the risk of tooth fracture. Hence, irrigants should be cautiously chosen to maximize efficiency and minimize adverse impacts on root canal dentin [47].

In the evaluation of the physical properties of root dentin different parameters should be considered including roughness, hardness, and fracture resistance [16, 28, 48]. Hardness refers to the ability of a solid material to withstand plastic deformation, elastic deformation, and destruction. Teeth hardness is classified into static and dynamic. The most commonly utilized technique for characterization is static indentation hardness which includes Knoop hardness, Vickers hardness, and nano-hardness [49].

The effects and penetration depth of therapeutic materials used on dentin during root canal preparation depend on the diameter and number of dentinal tubules [50]. Moreover, the tubular density is inversely related to microhardness, as it increases towards the pulp chamber [51]. Therefore, in the present study, dentin microhardness was measured in different root canal thirds of radicular dentin. The dentin microhardness is also affected by the extent of mineralization and the presence of hydroxyapatite in the intertubular substance [31].

The current findings revealed that the baseline microhardness was higher in the coronal third compared to the middle and apical thirds. The mechanical behavior is significantly attributed to the mineral content [52]. The more condensed mineral content in the coronal third may be responsible for the greater baseline microhardness. This higher mineral content makes dentin more resilient and capable of resisting local deformation. This study showed that normal saline did not significantly affect the VHNs, which is consistent with previous studies that used normal saline as a negative control [29, 47, 53].

Organic materials (chiefly collagen type I) play an important mechanical role in dentin, accounting for 22% of its composition. NaOCl triggers the depletion of the organic phase, which can lead to mechanical alterations [54]. The present findings indicate that immersion in NaOCl caused a decrease in dentin microhardness compared to the pre-treatment values, with a statistically significant effect in the coronal zone of the root canal dentin. This may be due to the breakdown of long peptide chains and chlorination protein terminal groups by NaOCl, leading to the formation of other species [55].

Previous studies have extensively investigated the effect of NaOCl on dentin microhardness [27–29, 31, 36, 50, 55, 56]. Consistent with the present study, some studies have reported a significant decrease in dentin microhardness after exposure to 2.5% NaOCl [27, 36, 55]. Bosaid et al. [56] detected that a 5-minute application of 1.5% NaOCl decreased the dentin microhardness, although it was statistically insignificant. In contrast, Philip et al. [55] detected that 2.5% NaOCl significantly decreased dentin microhardness in the apical third compared to the middle and cervical thirds. This reduction was justified by the low surface tension of NaOCl, which allowed it to penetrate long and narrow dentinal tubules through capillary forces or by diffusion into the dentin.

In the current study, the primary concentration of NPs solution before the experiment was set as 1024 µg/mL since we intended to investigate whether different NPs in their highest effective antimicrobial concentration would affect other characteristics of a good irrigant, such as microhardness. Silver nanoparticles are known as a popular root canal irrigant because of their antibacterial properties and low toxicity on human cells [17, 34, 57, 58]. However, the present study found that using them as a root canal irrigant reduced the root dentin microhardness at all thirds of the root canal. In agreement with the current study, Suzuki et al. [59] reported that AgNPs nanoparticles caused little to no alterations in the mechanical properties of dentin and resin cement at different root canal thirds. On the contrary, Hassan et al. [60] observed that using AgNPs nanoparticles as an intracanal medicament gradually increased the microhardness of root canal dentin.

Baras et al. [61] assessed an innovative root canal sealer incorporating dimethylaminohexadecyl methacrylate (DMAHDM), AgNPs nanoparticles, and amorphous calcium phosphate nanoparticles, and detected that this sealer did not jeopardize the physical structure of root dentin such as hardness. On the other hand, Jowkar et al. [26] demonstrated that using AgNPs nanoparticles as the final irrigant improved the fracture resistance of teeth with previous endodontic treatment.

The present study and some previous ones [16, 62] showed that positively-charged AgNPs nanoparticles can enhance the mechanical properties of dentin. However, standard AgNPs nanoparticles used in this study exhibited opposing effects compared to the positively-charged variety (Im-AgNPs nanoparticles), indicating that the synthesis method can impact nanoparticles properties [63]. Possibly, the decrease in microhardness following irrigation with AgNPs irrigant is due to their generally negative surface charge, which can interfere with material properties.

To date, no previous research has investigated the impact of Im-AgNPs nanobased irrigant on root dentin microhardness. Im-AgNPs nanoparticles have demonstrated superior antibacterial activity, even at lower concentrations, when compared to chlorhexidine and NaOCl [15]. Farshad et al. [16] reported that Im-AgNPs nanoparticles modified the physicochemical properties of dentin and enhanced the roughness of root dentin. The underlying reasons were either due to the ionic liquid (imidazole) nature or the discrepancy in charge distribution on the cationic part of the imidazole and dentin surface.

As the first investigation into the effect of ZnONPs nanoparticles on dentin microhardness, the current findings demonstrated that this irrigant significantly elevated the dentin microhardness at the coronal third of the root canal. Conversely, Im-AgNPs nanoparticles significantly increased the microhardness at the middle and apical parts of the root. This improvement in microhardness following the use of Im-AgNPs nanoparticles may be attributed to the nature of the ionic liquid (imidazole) or uneven charge distribution on the cationic part of the imidazole and dentin surface. Zhu et al. [64] reported that the incorporation of AgNPs and zinc nanoparticles with mesoporous calcium-silicate nanoparticles did not have any detrimental effects on the mechanical characteristics of dentin. The absence of hydroxyl ions in the aqueous solutions of these nanoparticles was mentioned as the probable cause of negative alterations in dentin’s mechanical properties.

In line with the present findings, Jowkar et al. [26] observed that ZnONPs nanoparticles enhanced root fracture resistance in endodontically-treated teeth. Although they found no significant difference between the ZnONPs and AgNPs, they reported that AgNPs nanoparticles had a greater positive effect on root fracture resistance compared to ZnONPs. Another research addressed the influence of incorporating AgNPs and ZnONPs nanoparticles into pit and fissure sealant on the microhardness of these sealants. The results showed that the microhardness of sealants containing AgNPs increased more significantly compared to those containing ZnONPs nanoparticles. The researchers suggested that the higher increase in microhardness could be attributed to the nature of the nanoparticles, with metals being harder than metal oxides even at lower concentrations [65]. Possibly the contrasts between our findings and those of previous studies were due to variations in the synthesis methods or concentrations of nanoparticles used.

The present study highlights the potential of Im-AgNPs and ZnONPs nanoparticles as alternative root canal irrigants, as they not only possess antibacterial properties, but also improve dentin microhardness. However, more in-vivo and in-vitro studies are required to fully evaluate the efficacy of these novel nanobased irrigants, including their effect on other dentin properties such as roughness and fracture resistance, and at different distances from the pulp–dentine interface.

One major limitation of ex-vivo studies like this is the potential variation in teeth studied, including age differences, which could affect the physicochemical characteristics of the root canal dentin.

## Conclusion

In conclusion, the use of root canal irrigants containing Im-AgNPs and ZnONPs nanoparticles can significantly improve the microhardness of the root canal dentin in the coronal (ZnONPs), middle, and apical thirds (Im-AgNPs).

## Data Availability

The datasets used and/or analysed during the current study are available from the corresponding author on reasonable request.

## List of Abbreviations

Zinc oxide: ZnONPs
Silver: AgNPs
Imidazolium-based silver: Im-AgNPs
Vickers Hardness Number: VHN
Sodium hypochlorite: NaOCl
Ethylenediaminetetraacetic acid: EDTA
Silver nitrate: AgNPsNO_3_
Transmission electron microscopy: TEM
X-ray diffraction spectroscopy: XRD
Energy-dispersive X-ray spectroscopy: EDS
Dimethylaminohexadecyl methacrylate: DMAHDM
